# New Appliances and Things Medical

**Published:** 1904-05-28

**Authors:** 


### NEW APPLIANCES AND THINGS MEDICAL.

[We shall be glad to receive, at our Office, 28 & 29 Southampton Street, Strand, London, W.C., from the manufacturers, specimens of all new preparations and appliances which may be brought out from time to time.]

### EVAPORATED CREAM—UNSWEETENED FULL-CREAM MILK.

(Fussell and Co., 4 Monument Street, London, E.C.)

This company's cream was noticed in The Hospital of January 23rd, 1904, and was found to be a thoroughly to be recommended sterilised cream free from preservatives. The full name of the product at present under consideration is that given above, but on the tins it is described as "The Silon Butterfly Brand Pure Unsweetened Evaporated Cream." It is not really cream, but is pure full-cream milk evaporated to the consistency of cream. Its certified chemical analysis gives the following figures:—Fat, 11·8 per cent.; casein and milk sugar, 24·7 per cent.; mineral matter, 2·1 per cent.; corresponding to the average percentage composition of a three-fold concentrated cows' milk. The milk has been sterilised and concentrated by evaporation; it gives no evidence of containing preservatives, and, either in its undiluted condition as a substitute for cream or diluted with three times its volume of water for fresh milk, has a pleasant flavour and gives no evidence of the manipulation to which it has been subjected. We think this article, like the cream, is to be recommended, and a great advantage it possesses is that it is supplied in small tins at the price of 2½d., so that waste is minimised.

